# Gut microbiota: effect of pubertal status

**DOI:** 10.1186/s12866-020-02021-0

**Published:** 2020-11-03

**Authors:** Xin Yuan, Ruimin Chen, Ying Zhang, Xiangquan Lin, Xiaohong Yang

**Affiliations:** Department of Endocrinology, Genetics and Metabolism, Fuzhou Children’s Hospital of Fujian Medical University, NO.145, 817 Middle Road, Fuzhou, 350005 China

**Keywords:** Puberty, Children, Adolescent, 16 s rRNA, Gut microbiota

## Abstract

**Background:**

The make-up of gut microbiota at different puberty stages has not been reported. This cross-sectional study analyzed the bio-diversity of gut microbiota at different puberty stages.

**Result:**

The subjects (aged 5–15 years) were divided into non-pubertal (*n* = 42, male%: 66.7%) or pubertal groups (*n* = 47, male%:44.68); in both groups, *Firmicutes*, *Bacteroidetes* and *Proteobacteria* were the dominant phylum. There was no difference of alpha- and beta-diversity among disparate puberty stages. Non-pubertal subjects had members of the order Clostridiales, family *Clostridiaceae*, genus *Coprobacillus* which were significantly more prevalent than puberty subjects. Also, the pubertal subjects had members of class *Betaproteobacteria*, order *Burkholderiales* which were significantly more prevalent than the non-pubertal subjects. Their relative abundance was independent of BMI-Z. In the pubertal subjects, the abundance of genus *Adlercreutzia*, *Ruminococcus, Dorea, Clostridium* and *Parabacteroides* was associated with the level of testosterone.

**Conclusions:**

This is the first report of the diversity of gut microbiota at different puberty stages. The various species of gut microbiota changed gradually associated with puberty stages. Differences in gut microflora at different pubertal status may be related to androgen levels.

**Supplementary Information:**

**Supplementary information** accompanies this paper at 10.1186/s12866-020-02021-0.

## Background

Puberty constitutes an important phase of life, which is associated with profound physiological changes related to sexual maturation during the transition toward adulthood. These somatic developmental changes are predominantly driven by hormones and are accompanied by psychological adjustment. Therefore, this dynamic period represents an unparalleled opportunity to assess potential hormonal impacts on gut microbiota [1]. Previous studies pointed out that human gut microbiota was adult-like after the first 3 years of life and were relatively stable [2, 3]. However, although healthy pre-adolescent children (ages 7–12 years) and adults harbored similar numbers of taxa and functional genes, their relative composition differed significantly [4]. Nonetheless, Enck et al. conducted a large-scale study, using conventional colony plating to assess numbers of several bacterial genera, found no noticeable changes in children between 2 and 18 years old [5]. Recently, with the expansion and availability of bacterial DNA sequencing technology, a study compared the distal intestinal microbiota composition between adolescents (11–18 years of age) and adults, founding the abundance of the genera *Bifidobacterium* and *Clostridium* was statistically significant higher in adolescent samples compared with adult samples [6].

Every organ is affected by the extensive changes in circulating hormone during puberty [7, 8]. We postulated that these marked changes in hormone levels would alter the intestinal flora. No previous microbiota study has explored the full span of growth from pre-puberty to late puberty. Such information is instructive given the association between gut microbiota during growth and adult disease risk [9]. To this end, we utilized 16 s rRNA gene sequencing to compare fecal microbiota profiles from pre-puberty to adolescence, ranging in age from 5 to 15 years.

## Results

### Study subjects

The mean age of the 89 participates was 9.75 ± 1.92 years (ranging from 5.5 to 14.3 years) and 55.06% were boys. The majority (73.03%) were obese based on BMI, and 26.97% had normal BMI.

Based on puberty status, the subjects were divided into non-pubertal group (*n* = 42, 66.7% male) and pubertal group (*n* = 47, 44.68% male). The average age was 8.36 ± 1.64 years and 10.99 ± 1.15 years, respectively. There was no statistical difference in BMI-Z scores or dietary habits between the two groups (*p* = 0.783 and 0.641, respectively). The non-pubertal group was further subdivided into a younger children group (*n* = 18, 66.7% male) and pre-pubertal group (*n* = 24, 66.7% male). And the pubertal group classified as early (*n* = 18, 77.8% male), middle (*n* = 14, 35.7% male), late (*n* = 15, 13.3% male). Of the 40 girls, 21 had E2 measured, 2 were non-pubertal with a level of E2 < 5 pg/ml, and 19 were pubertal with a level of E2 33.68 ± 35.80 pg/ml; 21 of the 49 boys had T measured, 6 were non-pubertal with a level of T 5.10 ± 4.16 ng/dl, and 15 were pubertal with a level of T 83.20 ± 98.55 ng/dl. There was no statistical difference in BMI-Z scores, mode of birth, feeding patterns or dietary habits among the groups (*p* > 0.05). Tables 1, 2 and Table S1, S2 describes the characteristics of the subjects.

**Table 1:** Clinical characteristics of the study population divided by puberty status

	Non-puberty (*n* = 42)	Puberty (*n* = 47	*P* value
Age (years)	8.36 ± 1.64	10.99 ± 1.15	< 0.001
Gender (male%)	66.7%	44.68%	0.054
Height (cm)	132.94 ± 10.62	148.96 ± 8.03	< 0.001
Weight (kg)	39.72 ± 15.53	54.09 ± 13.57	< 0.001
BMI (kg/cm^2^)	21.70 ± 5.56	24.07 ± 4.40	0.028
BMI-Z	1.92 ± 1.79	2.01 ± 1.13	0.783

**Table 2:** Clinical characteristics of the study population divided by puberty stages

	Younger children (5-8 years-old)	Pre-puberty	Early puberty	Middle puberty	Late puberty	*P* value
N (n)	18	24	18	14	15	
Age (years)	6.81 ± 0.74	9.53 ± 1.05	10.76 ± 0.95	10.85 ± 1.41	11.40 ± 1.05	< 0.001
Gender (male%)	66.7%	66.7%	77.8%	35.7%	13.3%	0.001
Height (cm)	124.64 ± 7.55	139.16 ± 8.05	148.35 ± 8.06	149.64 ± 9.84	149.06 ± 6.50	< 0.001
Weight (kg)	30.02 ± 9.17	47.00 ± 15.46	56.83 ± 12.53	55.55 ± 15.77	49.45 ± 12.18	< 0.001
BMI (kg/cm^2^)	18.98 ± 4.27	23.74 ± 5.61	25.53 ± 4.21	24.28 ± 4.31	22.12 ± 4.26	0.001
BMI-Z	1.47 ± 1.96	2.26 ± 1.61	2.26 ± 1.08	2.19 ± 1.02	1.55 ± 1.21	0.256

### Core microbiota in all the subjects

**Fig. 1:**
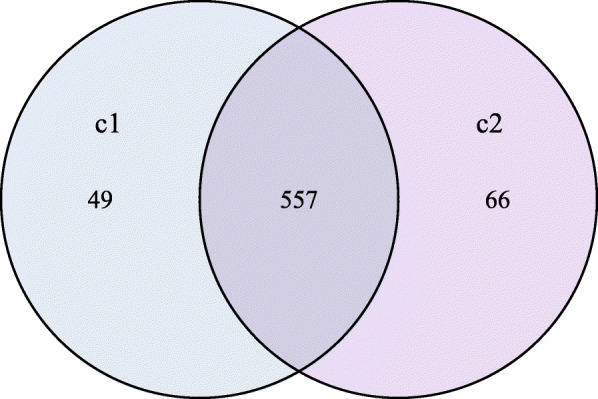
Shared OTU across the non-puberty and puberty groups. Different colors represent different groups, the interior of each circle represents the number of observed OTUs in the certain group. The overlapping area or intersection represents the set of OTU commonly present in the counterpart groups. Likewise, the single-layer zone represents the number of OTUs uniquely found in the certain group. C1: puberty group; C2: non-puberty group

**Fig. 2:**
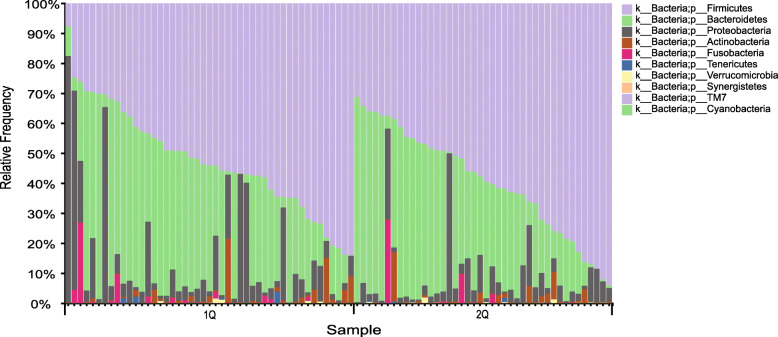
The taxa-bar of gut microbiota in non-pubertal and pubertal groups at phylum level

With 16S rRNA gene sequencing, 671 discrete bacterial taxa (OTUs) were identified. Most of these species belonged to the “shared” category of those common to multiple but not all samples. We also identified a “core” of 557 species shared among all fecal samples. The non-puberty group had 49 unique species and the puberty group had 66 unique species (Fig. 1). The core microbiota was dominated by phylum *Firmicutes*, *Bacteroidetes* and *Proteobacteria* in both the non-pubertal and pubertal groups (Fig. 2 and Table S3).

### Microbiota profiles with different puberty status

#### OTU classification

**Fig. 3:**
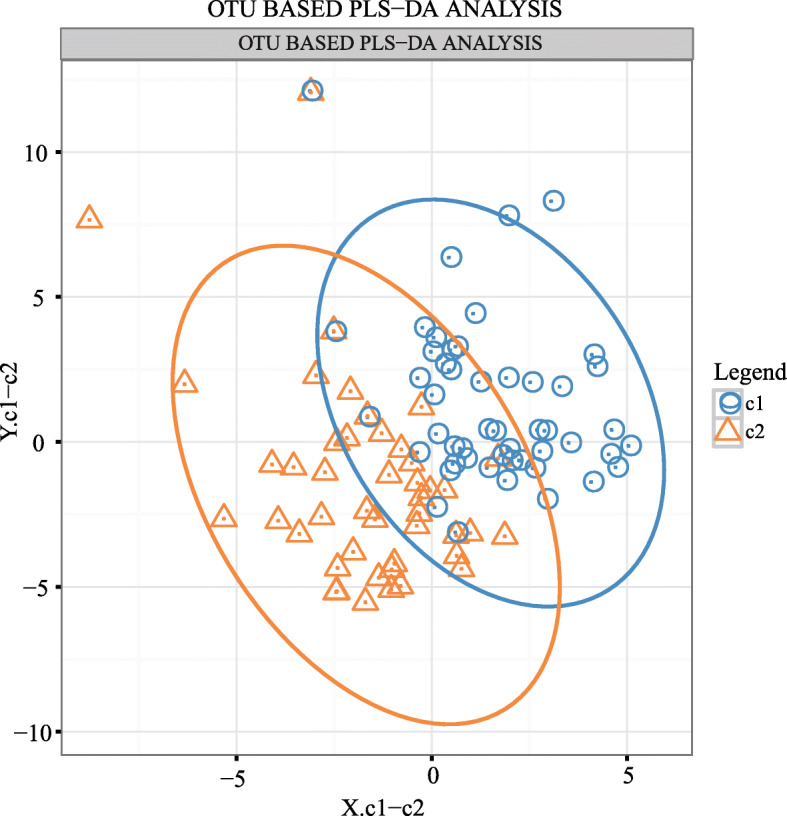
PLS-DA based on OTU abundance. The horizontal axis and the vertical axis indicate the top 2 components. Each dot denotes one sample. Samples are colored and grouped by elipse according to their group information. C1: puberty group; C2: non-puberty group

Using PLS-DA, the non-pubertal and pubertal groups can be distinguished to a certain extent, suggesting that the two groups differed in the classification of the gut microbiota (Fig. 3).

#### Alpha- and beta-diversity

Regarding alpha-diversity, there was no significant difference of the Shannon diversity index, Observed OTUs, Faith’s phylogenetic diversity and Pielou’s evenness based on OTU distribution between non-pubertal and pubertal groups (all *p* > 0.05, Table S4).

**Fig. 4:**
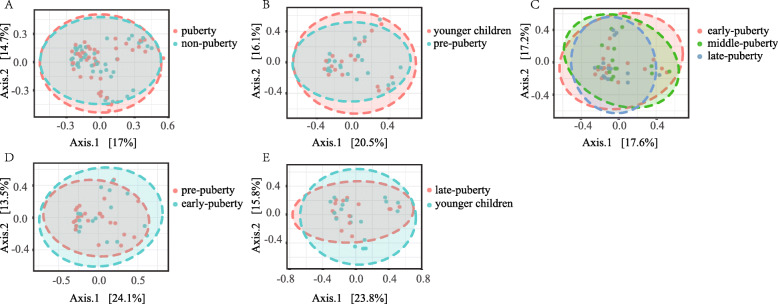
Principal coordinates analysis (PCoA) plot of pubertal and non-pubertal subjects (**a**), younger children and pre-pubertal subjects (**b**), early-, middle-, late-pubertal subjects (**c**), pre-pubertal and early-pubertal subjects (**d**), and younger children and late-pubertal subjects (**e**). The plots show the first two principal coordinates (axes) for PCoA using Bray-Curtis Distance method, and Statistical method PERMANOVA

Beta-diversity also did not differ significantly between these two aforementioned groups after correction for multiple testing (Table S5). By Distance method Bray-Curtis and Statistical method PERMANOVA, PCoA analysis illustrated that the gut microbiota samples from the non-pubertal group could not distinguish from the pubertal group [PERMANOVA] F-value: 0.712; R-squared: 0.008; *p*-value < 0.735 (Fig. 4a).

#### Bacterial taxa differences in subjects with different puberty status

**Fig. 5:**
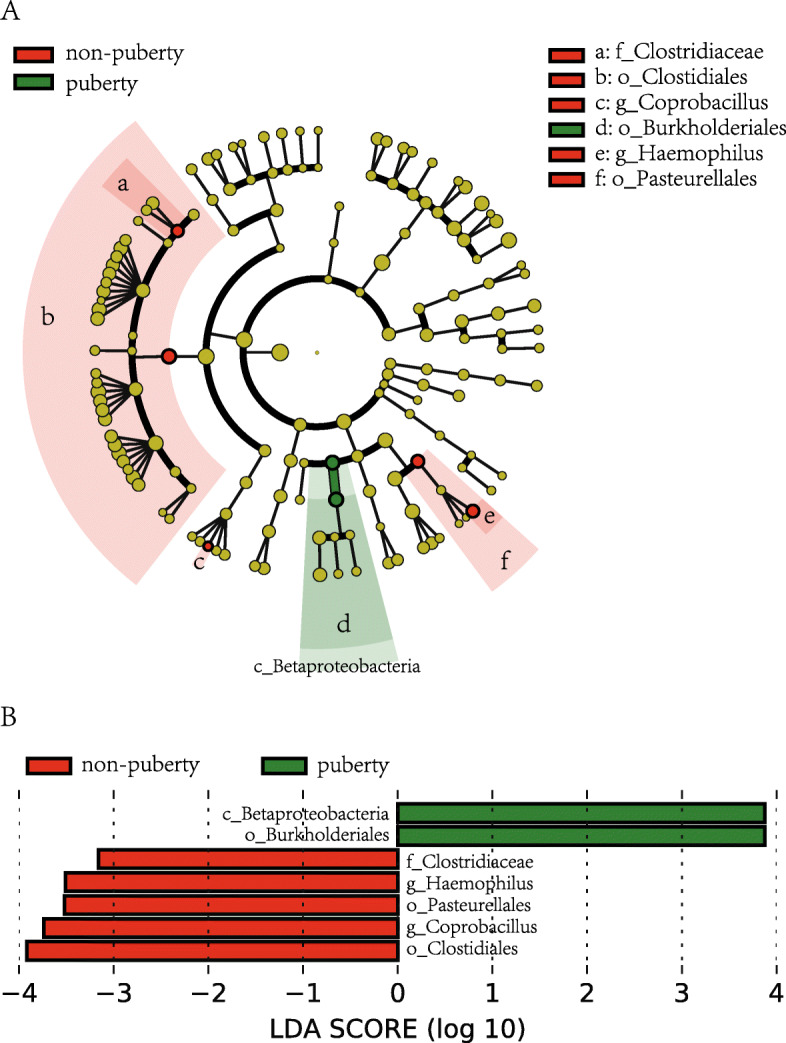
Differential biomarkers associated with genders in pubertal subjects and non-pubertal subjects. A linear discriminant effect size (LeFse) analysis have been performed (α value = 0.05, logarithmic LDA score threshold = 2.0)

We used Lefse analysis to identify bacteria where the relative abundance was significantly increased or decreased in each phenotypic category. Non-pubertal subjects had members of the Non-pubertal subjects had members of the order *Clostridiales*, *Pasteurellales*, family *Clostridiaceae*, genus *Coprobacillus* and *Haemophilus* that were significantly more prevalent than puberty subjects. Also, the pubertal subjects had members of class *Betaproteobacteria,* order *Burkholderiales* that were significantly more prevalent than the non-pubertal subjects (Fig. 5).

Spearman correlation analysis was used to detect an impact of BMI-Z on class *Betaproteobacteria*, order *Clostridiales, Pasteurellales, Burkholderiales,* family *Clostridiaceae,* genus *Coprobacillus* and *Haemophilus.* The results showed the relative abundance of order *Pasteurellales* and genus *Haemophilus* correlate with BMI-Z (*r* = 0.223 and 0.222, *p* = 0.036 and 0.036, respectively), whereas other differential bacterial taxa had no association with BMI-Z (*p* > 0.05).

### Microbiota profiles during puberty transition

#### Alpha-, beta-diversity and bacterial taxa differences in non-puberty subgroups

As for the alpha-diversity between younger children and pre-pubertal groups, there was no statistical difference of the Shannon diversity index, observed OTUs, Faith’s phylogenetic diversity and Pielou’s evenness based on OTU distribution (all *p* > 0.05, Table S4).

Beta-diversity also did not differ significantly between these two groups (all p > 0.05, Table S5). By Distance method Bray-Curtis and Statistical method PERMANOVA, PCoA analysis illustrated that the gut microbiota samples from the pre-pubertal group could not separate partly from the younger children group [PERMANOVA] F-value: 0.389; R-squared: 0.0096; *p*-value < 0.982 (Fig. 4b).

Lefse analysis found no differential bacterial taxa between pre-puberty group and non-puberty group (*p* > 0.05).

#### Alpha-, beta-diversity and bacterial taxa differences in puberty subgroups

As for the alpha-diversity among the three subgroups at the different puberty stages, the Shannon diversity index, observed OTUs, Faith’s phylogenetic diversity and Pielou’s evenness based on OTU distribution, there was no significant differences (all *p* > 0.05, Table S4).

Beta-diversity also did not differ significantly among the three subgroups after correction for multiple testing (all *p* > 0.05, Table S5). By Distance method Bray-Curtis and Statistical method PERMANOVA, PCoA analysis illustrated that the gut microbiota samples from the three groups could not distinguish from each other group [PERMANOVA] F-value: 0.857; R-squared: 0.0375; *p*-value < 0.679 (Fig. 4c).

Lefse analysis found no differential bacterial taxa among early-, middle- and late- puberty group (*p* > 0.05).

#### Alpha-, beta-diversity in non-puberty and puberty subgroups

The Shannon diversity index, observed OTUs, Faith’s phylogenetic diversity and Pielou’s evenness based on OTU distribution did not reveal any significant difference between the pre-puberty and the early puberty groups. Comparing the younger children group and the late puberty group, the Alpha- diversity indexes did not differ, either (all *p* > 0.05, Table S4).

Beta-diversity also did not differ significantly between the pre-puberty and the early puberty groups, or the younger children group and the late puberty group. The results were non-significant after correction for multiple testing (all *p* > 0.05, Table S5). By Distance method Bray-Curtis and Statistical method PERMANOVA, PCoA analysis illustrated that the gut microbiota samples from the early puberty group could not distinguish from pre-puberty group [PERMANOVA] F-value: 0.70375; R-squared: 0.01729; *p*-value < 0.698, and the gut microbiota samples from the late puberty group could not distinguish from younger children group, either. [PERMANOVA] F-value: 0.67589; R-squared: 0.021338; p-value < 0.801 (Fig. 4d, e).

### Correlations between sex hormone and bacterial abundance

To evaluate correlations between bacteria and serum sex hormones (testosterone and estradiol), Spearman’s rank analysis was adopted. In the pubertal subjects, the abundance of genera *Adlercreutzia, Ruminococcus* and *Dorea* was positively associated with the level of testosterone (*r* = 0.371, 0.471 and 0.488, *p* = 0.040, 0.007 and 0.005, respectively), and the abundance of genera *Parabacteroides* and *Clostridium* was positively associated with the level of testosterone (*r* = − 0.424 and − 0.361, *p* = 0.017 and 0.046, respectively). There was no association between the bacterial abundance and serum estradiol (all *p* > 0.05).

## Discussion

Recent studies have yielded insight into the intricate interactions between age and the gut microbiota by 16S rRNA sequencing [10]. With increasing age, it has been observed a gradual and steady reduction in the population of aerobes and facultative anaerobes, in concert with a simultaneous upsurge in the number of anaerobes [5, 11]. Previous study reported that the adolescent microbiota was found to differ functionally from that of adults, expressing genes related to development and growth [7], whereas the adult microbiota was more associated with obesity and inflammation [3, 5]. However, the make-up of gut microbiota at different puberty stages were not described [12].

Previous research inferred that during childhood there was less diversity in the gut microbial community compared to adults [13]. In this study, we found no difference in alpha- or beta-diversity between non-pubertal and pubertal subjects. However, differential abundance testing showed differential bacterial taxa between non-pubertal and pubertal subjects. Few studies have addressed differences in gut microbiota as children age [14]. A recent high-throughput analysis of distal gut microbiota found that even though adolescents apparently share a core microbiota configuration with that of adults, they harbor a less complex and considerably different microbiota: Compared with adults, the abundance of *Clostridia*, *Bifidobacterium* and *Clostridium* genera were significantly higher in adolescents [2, 5, 6]. Furthermore, Paliy O et al. developed a microbiota microarray and found that compared with adults, fecal samples from children harbored more *Bacteroidetes* and *Proteobacteria*, and less *Clostridia*. A number of other putative differences were also reported at the genus level [15].

Specifically, this study found that Non-pubertal subjects had members of the order *Clostridiales,* family *Clostridiaceae*, genus *Coprobacillus* that were significantly more prevalent than puberty subjects. Also, the pubertal subjects had members of order *Burkholderiales* that were significantly more prevalent than the non-pubertal subjects, and the relative abundance of them were independent of BMI-Z. It has been reported that the abundance of gut *Coprobacillus* was enriched in BALB/c male mice [16], suggesting a possibility that the abundance of gut *Coprobacillus* could be affected by sex hormon*e*. Dong G reported that gut *Coprococcus* was enriched in girls with idiopathic central precocious puberty [17]. All microbes in genera *Coprococcus* promote SCFAs production [18–20]. Therefore, it is plausible that SCFAs-producing bacteria are increased in pubertal subjects to promote the expression of the leptin gene, which in turn activate the hypothalamic-pituitary-gonad axis, and lead to the onset of puberty [16]. However, we didn’t find the abundance of gut *Coprobacillus* changed between the pubertal and non-pubertal groups.

In addition to the bursts of gonadotropin-releasing hormone with puberty, many other circulating hormones come of age. These profound hormonal changes may be accompanied by changes in the composition of gut microbiota. In a study from the Netherlands, 61 children aged 2–18 years collected fecal samples weekly for 6 weeks, along with a follow-up sample after 18 months. The microbial composition stability varied per phylum at both short-term and long-term intervals. However, the age span of this study precluded the characterization of the gut microbiota at distinct puberty stages. We found there was no significant difference in alpha-, beta-diversity, or the differential bacterial taxa between the younger children and pre-pubertal groups. A study of Asian school-age children took the opinion that the presence and ratios of *Bifidobacterium* / *Bacteroides* and *Prevotella* enterotype-like clusters were closely associated with geographic regions, because of the varying diets and living environments [21].

Despite the limitations imposed by heterogeneity of the groups, the present study found a relationship between puberty and gut microbiota. During sexual development and growth through the adolescent period, the gut microbiota undergoes progressive changes, likely due to the hormonal surge or other age-related factors. Accordingly, the association between serum sex hormones and bacterial abundance was further analyzed. In the pubertal subjects, it was found that the abundance of genus *Adlercreutzia*, *Dorea, Clostridium* and *Parabacteroides* associated with the level of testosterone. A mouse model study showed that after inoculating germ-free C57BL/6 J mice with fecal bacteria from a man with short-term vegetarian and inulin-supplemented diet, the abundance of *Dorea* and *Clostridium* were over-represented in females [22]. Furthermore, Shin JH [23] reported that the abundance of *Dorea* correlated significantly with the level of testosterone in males, a finding consistent with the results of our study. As known that the two bacteria *Parabacteroides* and *Adlercreutzia* could metabolize phytoestrogens with generation of secondary molecules such as secoisolariciresinol, enterolactone, and equol [24], we speculate that the two bacteria might be affected by sex hormones. The association between these bacteria and androgen warrants further investigated.

As a counter-narrative, could the adaptive intestinal microbiome effect serum hormone concentrations or tissue responsiveness [25]? Little is known about the causal inter-relationships between gut microbiota and pubertal development. As differences in gut microbiota become more pronounced at puberty, sex hormones might play an important role in shaping the gut microbiota composition is terra incognita [26, 27]. Relevant to our study, transferring the gut microbiota from adult male mice to immature females could result in the levels of testosterone elevated comparable to males, and the recipient’s microbiota altered [1]. Interesting to ponder, does a similar phenomenon due to the microbiota exists in humans? It is plausible that microbiota-driven hormone effects are in play and potentially influenced by genetic, metabolic, or psychosocial factors.

This study revealed the diversity of gut microbiota at different puberty stages. However, considering the small sample size and gender differences, for example, between the number of male patients in the prepubertal group and more female patients in the post-pubertal group, render this study at risk of Type 2 error. Even though we found no significant difference of alpha- and beta-diversity between pubertal and non-pubertal groups, a longitudinal study with large sample size wherein the participants are followed over an extended period (pre-puberty to puberty) would confirm a dynamic change in gut microbiome before and after puberty.

## Conclusion

This study is the first report of the characteristics of fecal microbiota during the transitional stages of puberty. Non-pubertal subjects had members of the order *Clostridiales*, family *Clostridiaceae*, genus *Coprobacillus* that were significantly more prevalent than puberty subjects. Also, the pubertal subjects had members of class *Betaproteobacteria,* order *Burkholderiales* that were significantly more prevalent than the non-pubertal subjects. Their relative abundance was independent of BMI-Z In the pubertal subjects, it was found that the abundance of genera *Adlercreutzia*, *Dorea, Ruminococcus, Clostridium* and *Parabacteroides* was associated with the level of testosterone. The explanation for the differences in these gut microbiota, and their potential metabolic and hormonal impact, requires additional study.

## Methods

### Study population

This study consisted of 89 children, aged between 5 to 15 years, managed by Fuzhou Children’s Hospital of Fujian Medical University as previously described [28].

### Dietary assessment

A semi-quantitative food frequency questionnaire, developed according to the dietary habits of South China, were completed by all participants. Dietary habits during the preceding month were assessed as previously described [28].

### Clinical assessment

Weight and height collected with duplicate measurements on standardized equipment were used to calculate BMI. BMI-Z scores were calculated based on Li Hui et al’s reference values, and the diagnostic criteria for obesity or normal weight of Chinese children were as published [29]. Puberty stage was defined according to the Tanner scale by the professionally trained pediatric endocrinologists. Subjects were divided into non-pubertal and pubertal groups. The former group was further subdivided into younger children (5–8 years old) and pre-puberty (Tanner stage 1, > 8 years old) group, and the puberty group was sub-divided into early (Tanner 2), middle (Tanner 3) and late (Tanner 4 and 5) stages for multi-point analysis. All participants maintained their usual dietary habits at least 3 days before blood sampling, and blood samples were collected from each participant after 12 h of fasting. Blood samples were stored at − 80 °C and analyzed within 2 weeks. Serum levels of estradiol (E2) and testosterone (T) were measured by chemiluminescent immunoassays (IMMULITE 2000, Siemens Healthcare Diagnostics Products Limited, Germany) using specific reagents.

### Fecal sample collection and processing

The parents of all participants were asked to collect the stool samples in standard collection tubes, and then transported immediately (within 2 h) at room temperature for storage at − 80 °C until further analysis.

### Genomic DNA extraction and high throughput sequencing

MagPure Stool DNA KF kit B (Magen, China) were used for extracting the microbial community DNA following the manufacturer’s instructions, and Qubit® dsDNA BR Assay kit (Invitrogen, USA) with a Qubit Fluorometer was used for DNA quantified and the quality was checked by 1% agarose gel.

Variable regions V3-V4 of bacterial 16 s rRNA rDNA libraries were prepared by polymerase chain reaction and high-throughput sequencing was performed as previously described [28].

### Statistical analysis

The clinical characteristics of all individuals including anthropometric parameters, levels of E2 and T, and other characteristics were statistically calculated using independent samples t test, Mann-Whitney U test, Kruskal-Wallis test and chi-square test by the Statistical Package for the Social Sciences software version 23.0 (SPSS Inc. Chicago, IL, USA). The normality of the data was tested using the Kolmogorov-Smirnov test. Clinical data are expressed in means ± standard error. *P* < 0.05 was considered statistically significant.

Statistical analyses of 16 s rRNA sequencing data were performed on alpha- and beta- diversity measurements by software QIIME2 (v2019.7) [30] as previously described [28]. Kruskal-Wallis Test was used for two groups comparison. Based on the OTU abundance, OTU of each group was listed. Venn diagram was drawn by Venn Diagram of software R (v3.1.1), and the common and specific OTU ID were summarized. Partial least squares discrimination analysis (PLS-DA) completed by package ‘mixOmics’ of software R. Linear discriminant analysis effect size (LEfSe, v1.0) was used to elucidate significantly different relative abundances of bacterial taxa, associated with different pubertal status. These analyses are presented in a bar plot and the parameters set with an LDA score of 2.0 with LEfSe [31].

## Supplementary Information

**Additional file 1: Table S1.** Dietary habits of the study population divided by puberty status (Chi-square test). **Table S2**. Sex hormone concentration of the study population as per puberty stage. **Table S3.** The absolute abundance of gut microbiota in non-puberty and puberty groups at phylum level. **Table S4.** Comparison of alpha-diversity between different puberty staging groups. **Table S5.** Comparison of beta-diversity between different puberty staging groups.

## Data Availability

The original contributions presented in the study are publicly available. The raw sequence data reported in this paper have been deposited in the Genome Sequence Archive (Genomics, Proteomics & Bioinformatics 2017) in National Genomics Data Center (Nucleic Acids Res 2020), Beijing Institute of Genomics (China National Center for Bioinformation), Chinese Academy of Sciences, under accession number CRA003010 that are publicly accessible at https://bigd.big.ac.cn/gsa.

## Abbreviations

LEfSe: Linear discriminant analysis effect size
PLS-DA: Partial least squares discrimination analysis
