# Supplementary material for: Gut microbiota: effect of pubertal status
Source: BMC Microbiol. 2020 Nov 3;20:334. doi: 10.1186/s12866-020-02021-0 (PMC7640488; doi:10.1186/s12866-020-02021-0)
Supplement: Supplementary file 1 — Additional file 1: Table S1. Dietary habits of the study population divided by puberty status (Chi-square test). Table S2. Sex hormone concentration of the study population as per puberty stage. Table S3. The absolute abundance of gut microbiota in non-puberty and puberty groups at phylum level. Table S4. Comparison of alpha-diversity between different puberty staging groups. Table S5. Comparison of beta-diversity between different puberty staging groups. [file 12866_2020_2021_MOESM1_ESM.docx]

**Supplementary Material**

**Table S1** Dietary habits of the study population divided by puberty status (Chi-square test)

|  | pre-puberty  (n=42) | Puberty  (n=47) | P value |  |
| --- | --- | --- | --- | --- |
| **Daily intake and type of food** | | | | |
| Cereals and vegetable-based | 11 | 15 | 0.643 |  |
| Cereals and meat-based | 31 | 32 |  |  |
| **Intake frequency** | | | | |
| Three meals per day | 39 | 42 | 0.717 |  |
| Snacks besides meals | 3 | 5 |  |  |
| **Usual cooking methods** | | | | |
| Fried | 37 | 43 | 0.730 |  |
| Steamed | 5 | 4 |  |  |
| **Ingestion frequency of sugary drinks** | | | | |
| almost everyday | 5 | 1 | 0.250 |  |
| three times a week | 4 | 4 |  |  |
| occasionally | 15 | 23 |  |  |
| almost not | 18 | 19 |  |  |

S-Table 2 Sex hormone concentration of the study population as per puberty stage

|  | Younger children  (5-8years-old) | Pre-puberty | Early puberty | Middle puberty | Late puberty |
| --- | --- | --- | --- | --- | --- |
| Female  (n) | 1 | 1 | 6 | 4 | 9 |
| E2(pg/ml) | <5 | <5 | 16.84±16.38 | 34.99±41.87 | 44.33±41.44 |
| Male(n) | 2 | 4 | 10 | 4 | 1 |
| T(ng/dl) | 5.59±4.36 | 4.86±4.72 | 22.31±25.17 | 197.08±76.99 | 236.6 |

S-Table 3. The absolute abundance of gut microbiota in non-puberty and puberty groups at phylum level

|  | non-puberty | puberty |
| --- | --- | --- |
| Bacteria;Firmicutes | 315461 | 317847 |
| Bacteria;Bacteroidetes | 170951 | 192471 |
| Bacteria;Proteobacteria | 32136 | 85234 |
| Bacteria;Actinobacteria | 7141 | 10362 |
| Bacteria;Fusobacteria | 7817 | 6828 |
| Bacteria;Tenericutes | 475 | 1097 |
| Bacteria;Verrucomicrobia | 549 | 547 |
| Bacteria;Synergistetes | 56 | 47 |
| Bacteria;TM7 | 20 | 23 |
| Bacteria;Cyanobacteria | 25 | 0 |

S-Table 4 Comparison of alpha-diversity between different puberty staging groups

| groups | alpha-diversity index | H | P value |
| --- | --- | --- | --- |
| Puberty v.s. non-puberty groups | Shannon | 0.002 | 0.96 |
|  | Observed OTUs | 1.634 | 0.20 |
|  | Faith’s phylogenetic diversity | 2.39 | 0.12 |
|  | Pielou’s evenness | 0.131 | 0.71 |
| Early-puberty v.s. middle-puberty groups | Shannon | 0.092 | 0.76 |
|  | Observed OTUs | 0.023 | 0.88 |
|  | Faith’s phylogenetic diversity | 0.244 | 0.62 |
|  | Pielou’s evenness | 0.092 | 0.76 |
| middle-puberty v.s. late-puberty groups | Shannon | 0.322 | 0.57 |
|  | Observed OTUs | 1.289 | 0.26 |
|  | Faith’s phylogenetic diversity | 1.714 | 0.19 |
|  | Pielou’s evenness | 0.190 | 0.66 |
| Younger children v.s. pre-puberty groups | Shannon | 0.258 | 0.61 |
|  | Observed OTUs | 1.616 | 0.20 |
|  | Faith’s phylogenetic diversity | 0.661 | 0.42 |
|  | Pielou’s evenness | 0.093 | 0.76 |
| Younger children v.s. late-puberty groups | Shannon | 0.472 | 0.49 |
|  | Observed OTUs | 2.042 | 0.15 |
|  | Faith’s phylogenetic diversity | 1.601 | 0.21 |
|  | Pielou’s evenness | 0.294 | 0.59 |
| Pre-puberty v.s. early-puberty groups | Shannon | 0.581 | 0.45 |
|  | Observed OTUs | 1.489 | 0.22 |
|  | Faith’s phylogenetic diversity | 3.634 | 0.06 |
|  | Pielou’s evenness | 0.165 | 0.68 |
| Pre-puberty v.s. late-puberty groups | Shannon | 0.068 | 0.80 |
|  | Observed OTUs | 0.001 | 0.98 |
|  | Faith’s phylogenetic diversity | 0.041 | 0.84 |
|  | Pielou’s evenness | 0.188 | 0.67 |

S-Table 5 Comparison of beta-diversity between different puberty staging groups

| groups | beta-diversity index | pseudo-F | P value |
| --- | --- | --- | --- |
| Puberty v.s. non-puberty groups | Bray-Curtis distance | 0.922 | 0.62 |
|  | Jaccard distance | 1.113 | 0.18 |
|  | Unweighted-unifrac | 1.199 | 0.205 |
|  | Weighted-unifrac | 0.594 | 0.74 |
| Early-puberty v.s. middle-puberty groups | Bray-Curtis distance | 0.709 | 0.92 |
|  | Jaccard diatance | 0.860 | 0.86 |
|  | Unweighted-unifrac | 0.641 | 0.87 |
|  | Weighted-unifrac | 0.429 | 0.86 |
| middle-puberty v.s. late-puberty groups | Bray-Curtis distance | 0.873 | 0.69 |
|  | Jaccard diatance | 1.043 | 0.28 |
|  | Unweighted-unifrac | 0.934 | 0.52 |
|  | Weighted-unifrac | 1.378 | 0.21 |
| Younger children v.s. pre-puberty groups | Bray-Curtis distance | 0.686 | 0.97 |
|  | Jaccard diatance | 0.835 | 0.99 |
|  | Unweighted-unifrac | 0.506 | 0.99 |
|  | Weighted-unifrac | 0.089 | 1.00 |
| Younger children v.s. late-puberty groups | Bray-Curtis distance | 0.808 | 0.81 |
|  | Jaccard diatance | 0.929 | 0.81 |
|  | Unweighted-unifrac | 0.744 | 0.85 |
|  | Weighted-unifrac | 1.101 | 0.35 |
| Pre-puberty v.s. early-puberty groups | Bray-Curtis distance | 0.769 | 0.86 |
|  | Jaccard diatance | 1.042 | 0.27 |
|  | Unweighted-unifrac | 1.187 | 0.25 |
|  | Weighted-unifrac | 0.586 | 0.75 |
| Pre-puberty v.s. late-puberty groups | Bray-Curtis distance | 0.785 | 0.87 |
|  | Jaccard diatance | 1.039 | 0.26 |
|  | Unweighted-unifrac | 0.810 | 0.74 |
|  | Weighted-unifrac | 1.065 | 0.35 |
